# Cannabis Use Disorder in Young Adults with Acute Myocardial Infarction: Trend Inpatient Study from 2010 to 2014 in the United States

**DOI:** 10.7759/cureus.3241

**Published:** 2018-08-31

**Authors:** Rikinkumar S Patel, Shailaja Reddy Katta, Riddhi Patel, Virendrasinh Ravat, Ravikumar Gudipalli, Viralkumar Patel, Jenil Patel

**Affiliations:** 1 Psychiatry, Griffin Memorial Hospital, Norman, USA; 2 Medicine, Garden City Hospital, Garden City, USA; 3 Epidemiology/Human Genetics and Environmental Sciences, The University of Texas School of Public Health, Houston, USA; 4 Infectious Disease, Clinical Infectious Disease Specialist, Las Vegas, USA; 5 Psychiatry, Magnolia Medical Clinic, Norcross, USA; 6 Internal Medicine, Blake Medical Center, Bradenton, USA

**Keywords:** cannabis, chronic marijuana abuse, recreational marijuana, heart attack, myocardial infaction, national trends, demographics, hospitalization cost, hospital stay, in-hospital mortality

## Abstract

Objective

This study determines the trend of acute myocardial infarction (AMI) in cannabis users. Demographic characteristics, hospitalization outcomes, and utilization of primary treatment modalities were evaluated in AMI inpatient population.

Methods

The study used data from the nationwide inpatient sample (NIS) for the years 2010-2014. We identified patients with AMI as the primary diagnosis (*N* = 379,843) and patients with cannabis use disorder as the secondary diagnosis. We used Pearson’s chi-square (*χ*2) test and independent sample t-test for measuring the categorical and continuous data, respectively.

Results

Inpatient admissions for AMI among cannabis users increased by 32% (*P* = 0.001). The overall mean age of cannabis users with AMI (41 years) remained stable with no significant differences observed across age groups. AMI was predominant in male cannabis users (79.1%), and there was a 38.3% increase in the prevalence in female cannabis users over five years (*P* < 0.001). About one-third of the cannabis users with AMI were covered by medicaid with a 70.5% pike (21% in 2010 to 37.5% in 2014; *P* < 0.001). There was a strong linear trend in nonelective admissions for AMI in cannabis users (*P* = 0.003) along with a moderate-to-severe morbidity (*P* = 0.001). Mean length of inpatient stay had a decreasing linear trend (*P* = 0.003), whereas hospitalization costs were increasing (*P* = 0.024), averaging $65,879 per admission for AMI. Cannabis users had a strong linear increasing trend (*P* = 0.007), with a 60% increase in in-hospital mortality (1.0% in 2010 to 1.6% in 2014).

Conclusion

Due to the risk of AMI, as seen in numerous case reports, the trend of emergency admission and severe morbidity due to AMI in cannabis users is also increasing. Also, cannabis users have a higher healthcare cost to manage AMI, yet the in-hospital mortality has risen tremendously over the last few years. It is imperative to know that chronic cannabis worsens the outcomes in AMI patients, and more clinical studies are needed to show the association of episodic use in cannabis abusers and AMI.

## Introduction

Cannabis is the most widely used illicit drug in the United States and, cannabis or marijuana smoking is rapidly increasing. It is well known that cannabis has several well-defined hemodynamic consequences, including a dose-dependent increase in heart rate, supine hypertension, postural hypotension, vasospasm, vasodilation and altered coronary flow to the heart and vascular system; however, whether it can trigger the onset of acute myocardial infarction (AMI) is unknown [1]. Although a few cases of AMI ascribing to cannabis or marijuana use have been published [2-6], little is known about the association between the cannabis smoking and AMI.

The risk of myocardial infarction is almost five-fold higher within the hour after cannabis smoking, although this heightened risk seems to decline rapidly beyond the first hour [7]. Thrombosis is due to the plaque rupture in a pro-coagulation environment: cannabis enhances oxidative stress with increase in the oxidized low-density lipoprotein formation, increase in the factor VII activity, activation and aggregation of platelets, and induction of an inflammatory response [8].

In a patient with cardiovascular risk, cannabis use seems to have a more pronounced effect on triggering angina than nicotine. A controlled cross-over study had compared effects of smoking tobacco and cannabis on angina pectoris and demonstrated that smoking one marijuana cigarette significantly decreased the exercise time until angina more than smoking one high-nicotine cigarette. Exercise time until angina showed a reduction of 50% after smoking a marijuana cigarette vs. 23% reduction after smoking one high-nicotine cigarette [9].

To the best of our knowledge, this is the first nationwide research to study the trend of AMI in cannabis users. Demographic characteristics, hospitalization outcomes, and utilization of primary treatment modalities were evaluated in these inpatients over the period of 2010–2014.

## Materials and methods

Data source

We used the nationwide inpatient sample (NIS) data from the healthcare cost and utilization project (HCUP) sponsored by the agency for healthcare research and quality (AHRQ) [10]. In the United States, the NIS has the largest inpatient database from a sampling of 4,411 nonfederal hospitals and covering 45 states. This represents a pool of approximately 20% of the US hospitals.

Sample population

In HCUP databases, more than 14,000 ICD-9-CM diagnosis codes and 3,900 procedure codes have been described which were further classified into a fewer number of categories by the AHRQ's clinical classification software (CCS) [11]. The sample population included patients aged 18–50 years. AMI was identified as the primary admitting diagnosis using CCS code 100 and cannabis use disorder (CUD) was identified using the validated International Classification of Diseases, 9th Revision, and Clinical Modification (ICD-9-CM) codes 304.30, 304.31, 304.32, and 305.2. These codes were used to identify individuals with cannabis abuse or dependence in our previous study [12]. 

Assessment of demographics and inpatient outcomes

To study the demographic trend in AMl patients with CUD over the period of 2010–2014, we included age, gender, race, income level, and insurance status. To measure the differences in hospitalization outcomes in the study population, the outcome variables of interest included severity of illness, which measures the loss of body functions and in-hospital mortality, which measures the number of inpatient deaths [10]. We calculated the length of inpatient stay as the number of nights the patient remained in the hospital for the management of AMI. Total charges of hospitalization do not include professional fees and noncovered charges [10]. The utilization of therapeutic procedures during hospitalization was identified using the CCS procedure codes [11] as follows; CCS code 44 for coronary artery bypass graft (CABG), CCS code 45 for percutaneous transluminal coronary angioplasty (PTCA), and CCS code 47 for coronary angiography.

Statistical analysis

We used Pearson’s chi-square (*χ*2) test and the independent sample *t*-test for measuring the categorical and continuous data, respectively. Furthermore, the categorical variables are in percentages. We also used discharge weight as provided in the NIS data [10], to obtain a nationally representative inpatient data. A *P* value lesser than 0.05 was used to determine the statistical significance of the test. Statistical analysis was done using the SPSS version 23 (IBM Corporation, New York) [13]. The NIS data does not contain any patients' personally identifiable information. So, the institutional review board (IRB) approval was not required for this study.

## Results

The total number of admissions with a primary diagnosis of AMI over the period 2010–2014 was 379,843 and 3.3% of these patients had a co-diagnosis of CUD. The proportion of AMI admissions involving CUD saw an increasing trend (*P* = 0.001) from 2198 (in 2010) to 2900 (in 2014), representing a 32% increase over five years.

The overall mean age of cannabis users with AMI (41 years) remained stable over the study period. This is due to no significant difference observed in younger age groups (18-35 years) and older age group of 36-50 years over the five years (*P* = 0.369). AMI was predominant in male cannabis users (79.1%), but with a decreasing trend. On the contrary, though AMI was seen in the lower proportion of females there was a 38.3% increase in the prevalence in female cannabis users from 16.2% in 2010 to 22.4% in 2014 (*P* < 0.001). Differences between races showed statistically nonsignificant trends, with Caucasians (52.6%) and African Americans (35.5%) having a variable trend, Hispanics (6.7%) having a decreasing trend, and an increasing trend seen in other races (Asians and native Americans).

About one-third of all the cannabis users with AMI had medicaid as the primary source of health insurance coverage with a 70.5% pike over the years (21% in 2010 to 37.5% in 2014; *P* < 0.001). Though self-payment also constituted 30% of overall primary payer source, there was a decreasing trend observed over the five-year period. In terms of median household income levels, the highest quartiles, i.e., patients below 25th percentile median household income, showed a variable trend, and the number of patients from the 26th to 50th percentile and 51st to 75th percentile income groups demonstrated a decreasing trend from 2010 to 2014 (*P* < 0.001). The demographic trends in AMI inpatient with CUD are mentioned in Table 1.

**Table 1:** Demographic trends in cannabis use disorder in the AMI inpatients. Significant *P* values < 0.05 at 95% confidence interval. AMI, acute myocardial infarction; CUD, cannabis use disorder.

Variable	2010	2011	2012	2013	2014	Total	P value for trend	Trend direction
Number of AMI admissions	80212	76576	75990	73440	73625			
Number of AMI admissions with CUD	2198	2375	2495	2625	2900	12593	0.001	Increasing
Prevalence of CUD	2.74%	3.10%	3.28%	3.57%	3.94%	3.32%	0.001	Increasing
Age at admission (in %)								
Mean age (in years)	41.04	41.00	40.94	41.03	41.23	41.05	0.428	Stable
18-35 years	22.9	24.8	23.4	23.2	22.6	23.4	0.369	Stable
36-50 years	77.1	75.2	76.6	76.8	77.4	76.6	0.369	Stable
Sex (in %)								
Male	83.8	81.1	77.2	77.0	77.6	79.1	<0.001	Decreasing
Female	16.2	18.9	22.8	23.0	22.4	20.9	<0.001	Increasing
Race (in %)								
Caucasian	53.1	50.8	52.7	52.4	53.7	52.6	0.754	Variable
African American	34.9	35.8	35.5	37.2	34.2	35.5	0.754	Variable
Hispanic	8.7	6.8	6.6	5.3	6.4	6.7	0.754	Decreasing
Native Americans/Asians	3.4	6.6	5.2	5.1	5.7	5.3	0.754	Increasing
Income level								
0-25th percentile	45.1	39.8	45.3	49.7	45.9	45.3	<0.001	Variable
26th-50th percentile	26.0	25.7	27.3	24.6	25.8	25.9	<0.001	Decreasing
51th-75th percentile	19.9	23.3	18.0	18.5	19.4	19.8	<0.001	Decreasing
76th-100th percentile	8.9	11.1	9.4	7.2	9.0	9.1	<0.001	Variable
Insurance								
Medicare	10.1	9.3	9.1	8.0	9.3	9.1	<0.001	Decreasing
Medicaid	22.0	26.7	32.7	29.4	37.5	30.1	<0.001	Increasing
Private	26.7	24.3	21.2	20.2	21.6	22.6	<0.001	Decreasing
Self-pay	32.7	31.4	29.6	32.8	25.6	30.2	<0.001	Decreasing
Other	8.5	8.3	7.5	9.5	5.9	7.9	<0.001	Decreasing

Majority of the cannabis users were admitted for AMI on an emergency or nonelective basis and there was a rising trend seen over the years (*P* = 0.003). The AMI patients with a moderate-to-severe morbidity due to loss of body function made up 68.6% of total admissions in cannabis users over the five-year period and showed a strong linear increasing trend (*P* = 0.001). Angiography was performed as a primary procedure in 22.8% cannabis users admitted for AMI and there was a stable trend over the years with a statistically nonsignificant difference. When there was a decreasing trend of use of PTCA in cannabis users, though a lower proportion of these patients were managed with invasive procedures like coronary artery bypass grafting (CABG), there was a rising trend of utilization of CABG from 2010 to 2014 (*P* = 0.092). Mean length of inpatient stay was 3.4 days and had a decreasing linear trend (*P* = 0.003), whereas hospitalization costs have been increasing (*P* = 0.024), averaging $65,879. Cannabis users had a strong linear increasing trend (*P* = 0.007), with a 60% increase in in-hospital mortality during inpatient management of AMI (1.0% in 2010 to 1.6% in 2014). Further hospital outcomes trends are described in Table 2.

**Table 2:** Outcome trends in cannabis use disorder in the AMI inpatients. Significant *P* values < 0.05 at 95% confidence interval. AMI, acute myocardial infarction; PTCA, percutaneous transluminal coronary angioplasty; CABG, coronary artery bypass grafting.

Variable	2010	2011	2012	2013	2014	Total	P value for trend	Trend direction
Admission (in %)								
Nonelective	92.8	94.6	97.0	96.0	94.5	95.0	0.003	Increasing
Elective	7.2	5.4	3.0	4.0	5.5	5.0	0.003	Decreasing
Severity of illness at admission (in %)								
Mild	34.3	31.8	30.1	34.7	27.4	31.5	0.001	Decreasing
Moderate	41.5	39.7	48.3	41.9	44.0	43.2	0.001	Increasing
Severe	24.2	28.5	21.6	23.4	28.6	25.4	0.001	Increasing
Treatment (in %)								
Angiography	22.2	22.2	22.8	24.8	22.2	22.8	0.447	Stable
PTCA	51.7	51.6	49.5	49.5	49.8	50.4	0.073	Decreasing
CABG	5.5	6.4	5.6	5.7	7.1	6.1	0.092	Increasing
Other outcomes								
Mean inpatient stay (in days)	3.72	3.39	3.32	3.13	3.39	3.38	0.003	Decreasing
Mean inpatient cost (in $)	62,327	62,803	62,023	67,676	72,968	65,879	0.024	Increasing
In-hospital mortality (in %)	1.0	1.0	1.0	1.7	1.6	1.3	0.007	Increasing

## Discussion

This study describes the analysis of CUD with AMI outcomes based on population-based hospital data over the period of 2010-2014. The study findings are supportive of what has been previously established in the literature about drug use and its direct impact on vascular function and myocardial infarction [7, 14]. The study showed a significant increase in the proportion of AMI admissions in the hospitals involving CUD as an associated risk factor. An increase in the number of visits by 32% over five years brings out an important public health implication, exaggerating the strong influence of CUD and its direct impact on AMI. These results agree with other key findings in the literature including several case reports and published articles [2, 3, 15]. 

We consider our study to be one of the few studies in the literature to assess the relationship between AMI and CUD with the type of insurance coverage used. A previous study by Rumalla et al. that focused on recreational marijuana use and subarachnoid hemorrhage was the only study that we observed comparable to our study criteria on the type of insurance covered. Rumalla et al. observed higher cannabis use among medicaid enrollees compared to nonusers (31.1% vs. 18.3%) [16]. In our study, 37.5% of cannabis users had medicaid insurance in 2014, which indicates an important problem of insurance being a supportive factor promoting CUD and thus increasing overall hospital burden in the United States. Along with this observation, it is also important to note that the 37.5% is also attributable to an overall 70.5% increase from the years 2010 to 2014, from 21% previously. Another important finding of this study was the increase in a number of emergency visits among cannabis users. This trend was found significant, along with an indication of 68% admissions in AMI patients attributable to cannabis use.

The study highlights the increase in overall healthcare costs and procedures in AMI patients pertaining to cannabis use specifically. This is mainly reflected by several important findings in our study viz. angiography being performed in 22.8% of cannabis users, 3.4 days’ mean length of stay, average hospitalization costs as much as $65,879, and above all, 60% increase in in-hospital mortality during inpatient management of AMI.

Our study also has several limitations. As this is among the pioneer studies assessing the type of insurance and its effect on AMI admissions, we did not have enough supporting literature to back up our findings. However, our study uses a stronger established dataset and high internal validity to report the findings, and thus sets a strong foundation with implications on the need for further research to warrant our findings. The second limitation was that we could not look at re-admission status for the participants, and that is because of the nature of the database. The database, however being a strong population-based registry with high generalizability sets a benchmark for these types of further studies, overweighs the importance of characteristic findings compared to limitations. However, this study had several strengths. First, our study exhibits a strong external validity in the form of generalizability of the results. Our second strength is that to our knowledge, this is the first study that assesses the healthcare insurance aspect and its impact on CUD and AMI admissions. The results of the study show an inadvertent increase in healthcare costs, mainly attributed to drug use and preventable risk factors that can be avoided. The final strength of the study is how the study avoids reporting bias, due to the use of the NIS dataset and its unique characteristic of data being coded independently of the practitioner.

## Conclusions

The prevalence of AMI in inpatient US population is decreasing, but the number of cases of AMI in cannabis users is rising. A linear increasing trend of AMI among cannabis users is seen in females, native Americans or Asians and those covered by medicaid. Due to the risk of AMI, as seen in numerous case reports, the trend of emergency admission and severe morbidity due to AMI in cannabis users is also increasing. Also, there was a spike seen in the utilization of more invasive procedures like CABG which may have indirectly increased hospitalization cost per inpatient management for AMI in cannabis users. Despite all these measures, the in-hospital mortality had risen tremendously over the last few years. It is imperative to know that chronic cannabis worsens the outcomes in AMI patients, and more clinical studies are needed to show the association of episodic use in cannabis abusers and AMI. Also, large-scale epidemiological studies are required to measure the prospective risks involved in cannabis abusers due to increasing cannabis or marijuana use for therapeutic purposes after medical marijuana laws are passed in the United States.
